# PIWI Proteins Are Dispensable for Mouse Somatic Development and Reprogramming of Fibroblasts into Pluripotent Stem Cells

**DOI:** 10.1371/journal.pone.0097821

**Published:** 2014-09-19

**Authors:** Ee-Chun Cheng, Dongwan Kang, Zhong Wang, Haifan Lin

**Affiliations:** 1 Yale Stem Cell Center and Department of Cell Biology, Yale University School of Medicine, New Haven, Connecticut, United States of America; 2 Genomics Division, Lawrence Berkeley National Laboratory, Berkeley, California, United States of America; National Institute of Environmental Health Sciences, United States of America

## Abstract

PIWI proteins play essential and conserved roles in germline development, including germline stem cell maintenance and meiosis. Because germline regulators such as OCT4, NANOG, and SOX2 are known to be potent factors that reprogram differentiated somatic cells into induced pluripotent stem cells (iPSCs), we investigated whether the PIWI protein family is involved in iPSC production. We find that all three mouse *Piwi* genes, *Miwi, Mili*, and *Miwi2*, are expressed in embryonic stem cells (ESCs) at higher levels than in fibroblasts, with *Mili* being the highest. However, mice lacking all three *Piwi* genes are viable and female fertile, and are only male sterile. Furthermore, embryonic fibroblasts derived from *Miwi/Mili/Miwi2* triple knockout embryos can be efficiently reprogrammed into iPS cells. These iPS cells expressed pluripotency markers and were capable of differentiating into all three germ layers in teratoma assays. Genome-wide expression profiling reveals that the triple knockout iPS cells are very similar to littermate control iPS cells. These results indicate that PIWI proteins are dispensable for direct reprogramming of mouse fibroblasts.

## Introduction

The germ cell is the totipotent cell type capable of generating an entirely new organism. Its extraordinary potential begins from the time of primordial germ cell (PGC) formation, with stage-dependent transcriptional reactivation of the pluripotency-associated gene network, followed by stepwise activation of PGC-specific genes [1]–[3]. Recent studies have shown that germ cell factors contribute to naive pluripotency in ESCs partly through the repression of differentiation and/or the integration into the core transcriptional regulatory network [4]–[6]. Multiple germline factors that function in PGC and/or spermatogonia, such as OCT4, SOX2, LIN28, PRDM14, and NANOG, are potent mediators of somatic cell reprogramming [4], [7]–[17]. In addition, *ex vivo* PGCs are able to give rise to pluripotent stem cells directly [18], [19]. All these observations have led to a notion that reprogramming of somatic cells to a ground state of pluripotency might entail a transition through a PGC-like state [20], [21], and that germ cell determinants may facilitate successful and efficient reprogramming of somatic cells into pluripotent stem cells.

We first discovered Piwi (*P*-element *i*nduced *wi*mpy testis) to be a critical factor in *Drosophila* germline stem cell self-renewal [22], [23]. In addition, the *Drosophila* Piwi protein is essential for the establishment of PGCs; depleting *piwi* leads to failure in PGC formation, while elevating *piwi* dose increases the number of PGCs [24]–[26]. Increasing evidence indicates that the PIWI protein family critically influences germline development from germline determination and stem cell maintenance to spermatogenesis across animal phylogeny [27], [28].

There are three PIWI proteins in mice, MIWI, MILI, and MIWI2, with individual mutants displaying unique defects during spermatogenesis. MIWI is expressed in male germ cells from the meiotic spermatocyte stage through the elongating spermatid stage and the mutant arrests at the round spermatid stage [29]. MILI is expressed from embryonic day 12.5 to the round spermatid stage [30]. Germline stem cells lacking MILI fail to self-renew or differentiate [30]. Occasionally, spermatogenic cells can escape the differentiation block but become arrested at the early pachytene stage of spermatogenesis [31]. MIWI2 is expressed in the embryonic and neonatal but not the adult testis. However, the terminal mutant phenotype of MIWI2 is observed much later during meiosis, with arrested leptotene spermatocytes and massive apoptosis of spermatogonia [32]. MIWI2 is a nuclear protein that may function epigenetically to set up a chromatin state in embryonic germ cells that is required for successful spermatogenesis in the adult [33].

Given the pivotal roles of PIWI family proteins in the germline, we investigated whether they can promote the generation and maintenance of iPSCs. Using mouse embryonic fibroblasts (MEFs) that are depleted for all murine PIWI family proteins, we showed that iPSC reprogramming can be achieved in the absence of all three PIWI proteins. The resulting cells exhibited pluripotent gene expression, were capable of differentiating into the three germ layers in teratoma assays, and had transcriptomes similar to those induced from littermate control cells containing wild type alleles of all three *Piwi* genes.

## Results

### *Piwi* genes are expressed in embryonic stem cells

**Figure 1:**
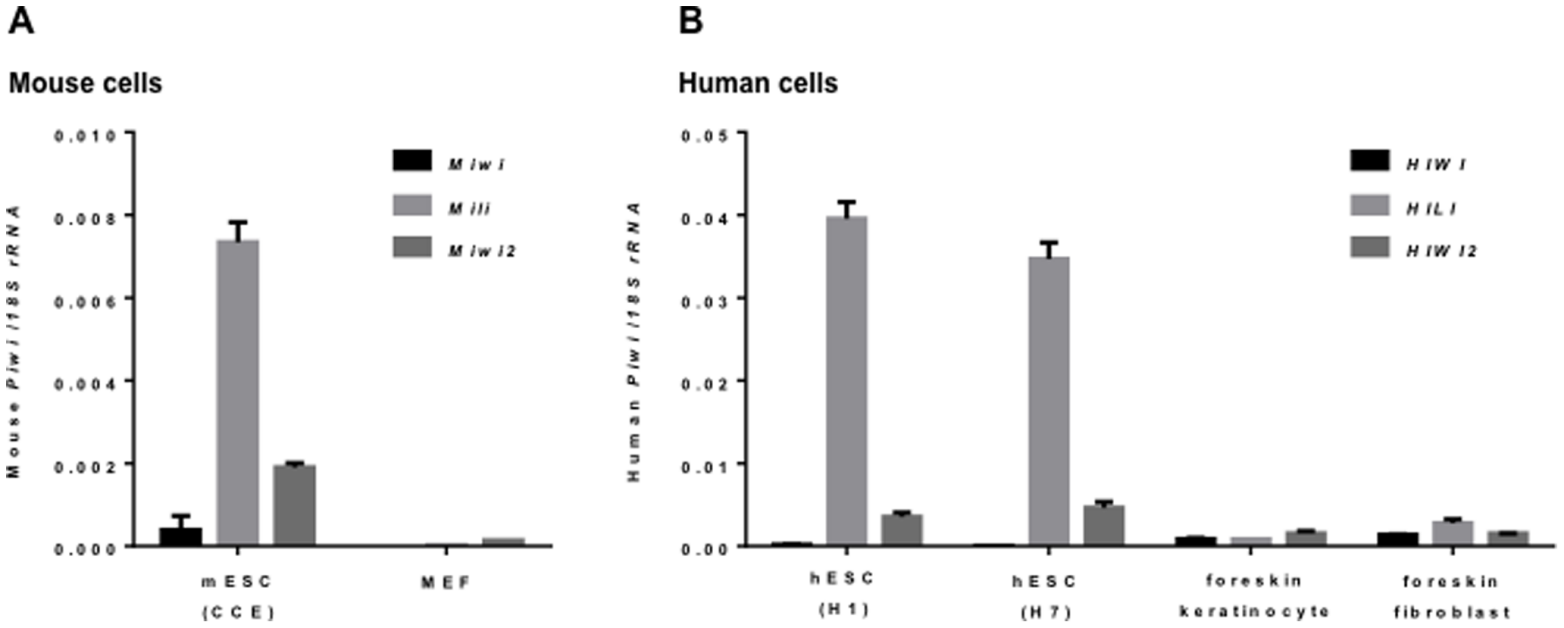
Expression of *Piwi* transcripts. qRT-PCR comparison of *piwi* expression in mouse cells (A) and human cells (B). RNA was isolated from mouse ESCs (CCE) and embryonic fibroblasts (MEF) and human ESCs (H1 and H7), human foreskin keratinocytes, human foreskin fibroblasts. The ratios of individual *piwi* genes/eukaryotic 18S rRNA are shown for both panels.

We first examined the gene expression patterns of *Miwi, Mili*, and *Miwi2* in mouse cells (Figure 1A), and *HIWI, HILI*, and *HIWI2* in human cells (Figure 1B). Quantitative RT-PCR analysis demonstrated that all of the *piwi* genes are expressed in ESCs with the exception of *HIWI*, which is almost undetectable. In contrast, the expression of all the genes in somatic cells is very low. The expression patterns of *piwi* family members are similar between mice and humans. Among the three *piwi* homologs, *piwil2* (*Mili* in mouse and *HILI* in human) transcripts were expressed at the highest level in ESCs. This indicates that *piwi* genes might be important for embryonic development.

### Mice lacking all mouse PIWI proteins are viable

To test the role of PIWI proteins in somatic development, we generated *Miwi*(-/-), *Mili*(-/-), and *Miwi2*(-/-) triple-knockout (TKO) mice (Materials and Methods) because individual *piwi* mutants are completely viable [29], [31], [32]. The *Miwi* mutant was generated by replacing almost the entire open reading frame (ORF) for MIWI with GFP, resulting in a fusion protein that contains only the first nine amino acid residues of MIWI fused to GFP via a PPRQ linker [29]. Thus, the *Miwi* mutant is a functionally null allele. In the *Miwi2* mutant, multiple in-frame stop codons were inserted, producing a protein-null allele [32]. The *Mili* mutant allele has exons 2 to 5 deleted, which corresponds to the first 209 amino acid resides of the MILI protein [31]. No MILI protein was detected by western blotting using an antibody against the conserved PAZ domain (386-494 amino acid residues), which is located downstream of the deletion [34]. This indicates that the *Mili* mutant used in this study is also protein-null. Thus, the triple mutant we generated completely has lost the function of all three *Piwi* genes in mice.

Despite this, the triple mutant females were all fertile up to 8 months and generated viable offspring (*n* = 12). This demonstrates that the PIWI-piRNA pathway is dispensable for both viability and fertility in female mice, which is in contrast to the requirement of the pathway in *Drosophila* for viability and female fertility [35]. We therefore bred TKO female mice to triple heterozygous males to generate TKO embryos and live mice. The offspring of these crosses (*n* = 120) displayed normal Mendelian frequencies (TKO expected frequency of 12.5% matched the observed frequency of 15.8%), suggesting that PIWI is not required for embryonic development or postnatal survival (Table 1). In addition, both male and female TKO mice were viable up to 10 months of age (*n* = 19), the longest time that we have tested, and appeared to be indistinguishable from heterozygous littermates, despite the defects in male germline development (data not shown). These results suggest that PIWI proteins are dispensable for normal mouse development and survival.

**Table 1:** Genotypes of 120 offspring at age 10 months from male triple heterozygous–female triple knockout mating.

Cross (male X female)	Offspring genotype (*Miwi, Mili, Miwi2*)							
Triple Het x TKO	+/−, +/−, +/−	−/−, +/−, +/−	+/−, −/−, +/−	+/−, +/−, −/−	−/−, −/−, +/−	+/−, −/−, −/−	−/−, +/−, −/−	−/−, −/−,−/−
**Observed number**	8	13	23	14	13	19	11	19
**Observed rate (%)**	6.7	10.8	19.2	11.7	10.8	15.8	9.2	15.8
**Expected rate (%)**	12.5	12.5	12.5	12.5	12.5	12.5	12.5	12.5

### PIWI is dispensable for generating iPS Cells

To assess whether PIWI proteins play a role in reprogramming differentiated somatic cells to induced pluripotent stem cells, we performed reprogramming experiments in Oct4-GFP mouse embryonic fibroblasts (MEFs) [36] that were derived from TKO embryos. We transduced the MEFs with retroviruses encoding OCT4, SOX2, KLF4, and c-MYC [7]. We also included the HDAC inhibitor VPA in some of these experiments, as this has been shown to enhance iPSC reprogramming efficiency [37]. We observed colonies approximately one week after virus transduction. Oct4-GFP+ colonies appeared at day 10.

**Figure 2:**
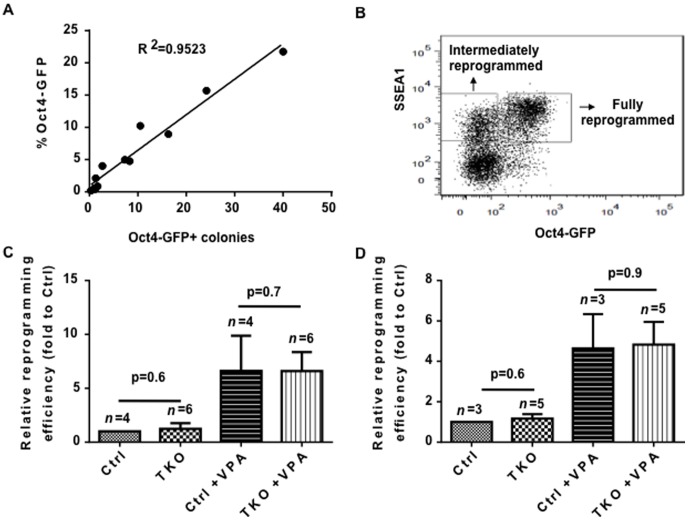
Reprogramming efficiency in PIWI-deficient MEFs is not compromised. (A) Correlation between GFP+ colony number and percentage 12 days post transduction. Each dot represents a single well of the reprogramming experiments. (B) Representative FACS plot for SSEA1/Oct4-GFP at 12 days post-viral transduction. (C, D) Relative reprogramming efficiencies are shown, with the fold changes indicated. (C) Fully reprogrammed efficiency, assessed by the percentage of SSEA1+Oct4-GFP+ cells; (D) Intermediately reprogrammed efficiency, assessed by percentage of SSEA1+Oct4-GFP- cells; Student's t-test (two-tailed) is used for statistics. Error bars, standard error. n = experiments with independent MEFs. Ctrl, wild type or heterozygous littermate controls; TKO, triple knockout of piwi.

To determine the reprogramming efficiency without bias, we first defined the time point when the number of manually counted GFP+ colonies correlates with the percentage GFP+ cells as determined by fluorescence-activated cell sorting (FACS) analysis. We found that at day 12 post retroviral infection, the number of GFP+ colonies correlated well with the FACS quantification of % GFP (R^2^ = 0.9523) (Figure 2A). Therefore, we tested the reprogramming efficiency at day 12 for all subsequent experiments. Reprogramming efficiencies were determined by FACS for expression of Oct4-GFP and SSEA1 staining, with Oct4-GFP+/SSEA1+ cells being indicative of fully reprogrammed cells. By contrast, Oct4-GFP-/SSEA1+ cells represent an intermediate step of reprogramming [36] (Figure 2B). We found that both fully and intermediately reprogramming efficiencies were not discernible between TKO MEFs and control (Ctrl) MEFs (Figure 2C and 2D). VPA, which is believed to act at the epigenetic level, increased the reprogramming efficiency by ten-fold in both TKO and Ctrl MEFs, indicating that PIWI proteins are not required for VPA enhancement of reprogramming efficiency.

**Figure 3:**
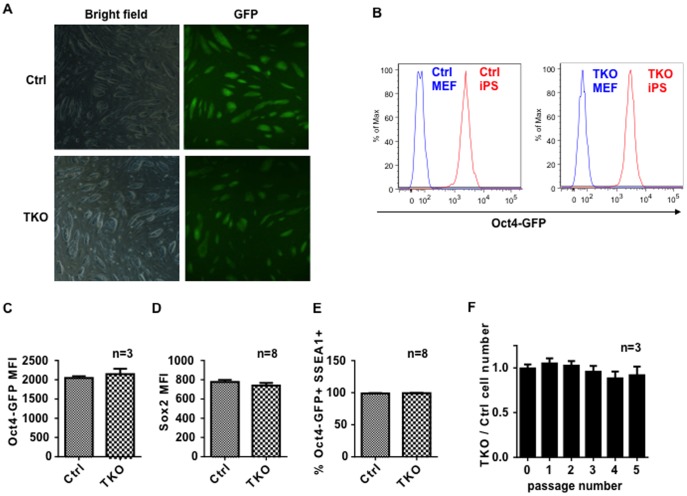
Piwi-deficiency does not affect reprogramming of MEFs. (A) iPS colonies exhibited typical ES cell morphology and expressed Oct4-GFP homogeneously. (B) Representative figure showing TKO iPS cells expressed comparable level of Oct4-GFP as Ctrl cells. (*n* = 20). Relative expression levels of (C) Oct4-GFP and (D) SOX2 proteins, as denoted by quantitative mean fluorescence intensity (MFI) shown by FACS analysis (*n* = 3 and 8, respectively). (E) Both TKO and Ctrl cells remained pluripotent and express Oct4-GFP^+^ SSEA1^+^ (>99%) over 30 passages. (F) A competition strategy was designed to determine if Piwi depletion compromises iPSC self-renewal. GFP+ cells (marked the iPSCs) were mixed in a 1-to-1 ratio with normal mouse ESCs (GFP-) cells, and cultured in the presence of LIF. With each passage the ratio of GFP/total cells was measured by FACS. The proportion of GFP+ cells with TKO were indiscernible from Ctrl cells over five passages (n = 3). Ctrl, wildtype or heterozygous littermate controls; TKO, triple knockout of piwi.

Furthermore, PIWI-deficient iPSCs were expandable, and showed morphology and proliferation similar to control iPSCs (Figure 3A). These cells expressed the pluripotency-associated factors OCT4 and SOX2 at comparable levels to those in control iPSCs (Figure 3B-3D), and could be maintained in an undifferentiated state over 30 passages (Figure 3E). To further test that iPSCs can be maintained without the PIWI protein family, we performed competitive cell growth assays [38]. In these assays, either TKO or Ctrl iPSCs expressing Oct4-GFP were co-cultured with a mouse ES cell line, S1B6A, at a 1-to-1 ratio (counted as passage 0). We monitored over five passages to determine if TKO exhibited any growth advantages or disadvantages. We anticipated that if PIWI proteins did not affect iPSC self-renewal, the growing populations of % GFP+ iPSCs would remain the same as Ctrl cells. Indeed, supporting the results that TKO iPSCs express robust pluripotency markers, the percentage of GFP-positive cells in TKO and Ctrl cells were indiscernible over five passages (Figure 3F).

**Figure 4:**
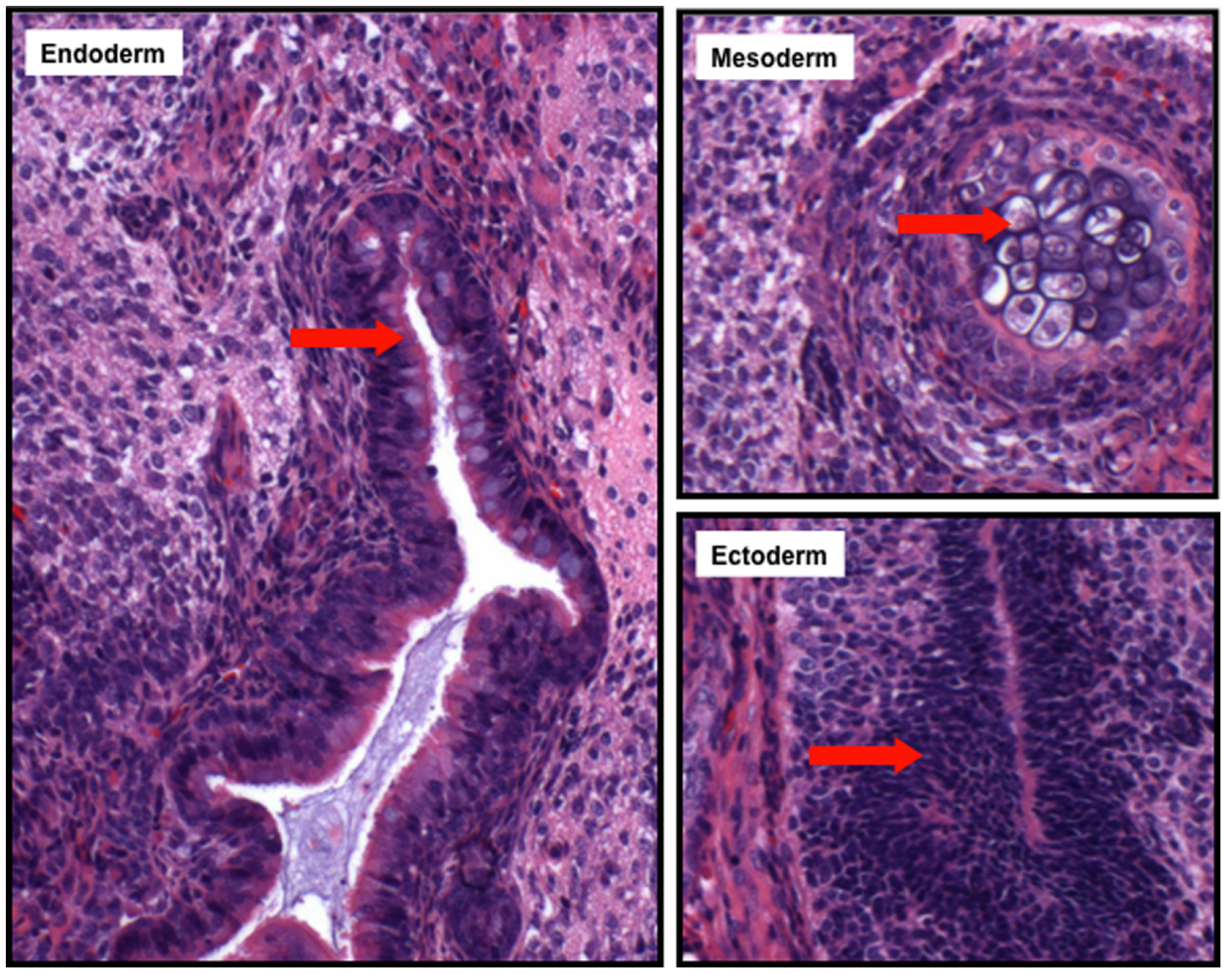
PIWI-deficient iPSCs form teratomas normally. Hematoxylin and eosin staining of teratoma sections showed differentiation of TKO iPS cells to tissues derived from all three germ layers, including the gut (endoderm), cartilage (mesoderm), and neural epithelium (ectoderm).

To assess their developmental potential, PIWI-deficient iPSCs were injected subcutaneously into SCID/Beige mice. The resulting teratomas displayed differentiated cells from all three germ layers (Figure 4). This observation is consistent with the finding that TKO knockout mice are able to develop normally into adulthood. Taken together, all of the above results indicate that reprogramming can occur equally efficiently in the absence of PIWI proteins.

### Global gene expression of iPSCs is largely unaffected by *Piwi* mutations

**Figure 5:**
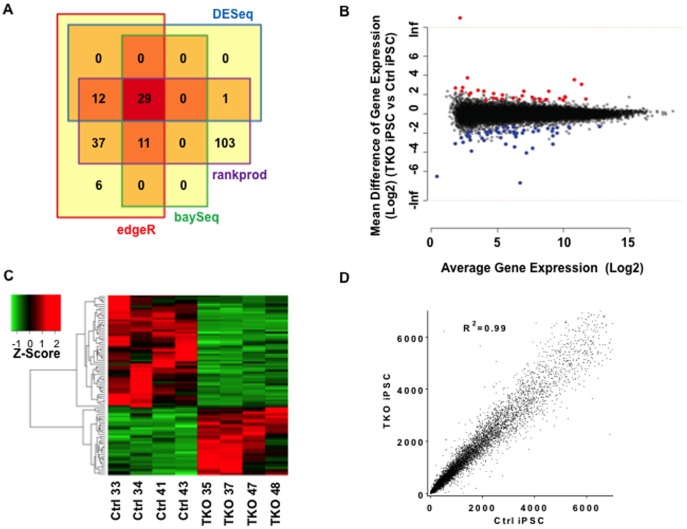
Differential expressed genes (DEGs) in PIWI-deficient iPSCs. (A) Venn diagram of the number of DEGs in 4 statistical methods. For the robustness of DEGs detection, we chose 90 DEGs which were identified at least two methods for further downstream analysis. (B) MA plot of all genes tested. X-axis represents average gene expression and Y-axis represents log fold changes. Red or blue dots represent up or down regulated genes, respectively. (C) Heatmap of 90 DEGs clustered by expression patterns. Each gene expression was standardized and the color represents z-score of each expression. Red or green color represent up or down regulation of the genes. (D) Correlation analysis of gene expression level between TKO and Ctrl iPSC (*n* = 4).

Next we investigated whether PIWI proteins are involved in regulating the gene expression of iPSCs by RNA-seq analysis of the transcriptomes of PIWI-deficient iPSCs (Figure 5 A–D). After normalization, the average counts of *Miwi, Mili*, and *Miwi2* in Ctrl iPSCs are 15, 895, and 594, respectively. This is consistent with our qRT-PCR expression pattern data, with *Mili* being the highest among the *piwi* family members in pluripotent cells. Direct comparison between TKO and Ctrl lines showed 90 differentially expressed genes (false discovery rate (FDR)-adjusted p-value<0.05 with >2.5 fold change in expression) (Table 2). Among these, 34 transcripts were up-regulated in TKO iPSCs, whereas 56 transcripts were down-regulated. Note that there was an up-regulation of *Mili (Piwil2)* and *Miwi2 (Piwil4)* transcripts in TKO iPSCs, but these were non-functional truncated transcripts, as described above. However, known pathways that are important to mESC self-renewal, such as LIF–gp130–Stat3, BMP–TGF-β–Smad, MAPK–ERK, and WNT were not affected [39]–[42]. The most well known factors for reprogramming, such as Oct3/4, Sox2, Klf4, Myc, Nanog, LIN28, and Glis1 [43], were also not affected by Piwi mutations. For functional annotation, we performed gene ontology (GO) analysis of the differentially regulated genes in TKO iPSCs. Top GO terms for the down-regulated genes were most involved in biosynthetic and metabolic process (Abra, Npas4, Sorbs1, Inhba, Epas1, LOC100048537, Fgf2, Ghrh) (Table S1 in File S1). Top GO terms for the up-regulated genes were most involved in membrane potential or neurogenesis (Olig1, Olig2, Grin1, Bnip3, Sema5a) (Table S2 in File S1). Correlation analysis between the TKO and Ctrl lines showed a strong R-value of 0.99 (Figure 5D). Since there was no detectable effect with a loss of PIWI proteins in these cells, it is likely that such small variations in gene expressions are routinely evident even between different wild-type iPSC lines. Although it is beyond the scope of this manuscript, it will be interesting to examine if TKO mice reveal any metabolic abnormalities or neurogenesis defects. Taken together, our results strongly indicate that PIWI-deficient pluripotent stem cells at this stage are highly similar to Ctrl iPSCs.

**Table 2:** Differentially expressed genes in triple knockout iPS cells.

Gen Bank ID	Symbol	Mean count in Ctrl	Mean count in TKO	Fold Change
NR_028101		1252.5	8.8	−143
NM_178730	Tmprss11f	13.0	0.1	−91.8
NM_013930	Aass	594.5	35.6	−16.7
NM_008096	Gc	31.5	2.1	−14.7
NR_033602		699.8	58.3	−12
NR_033599		257.0	24.1	−10.7
NR_015519		1752.5	195.8	−8.94
NM_183320	EG331529	147.1	16.5	−8.94
NM_010285	Ghrh	10.4	1.2	−8.56
NM_026108	Hdhd1a	14.8	1.9	−7.75
NM_029422	Tm7sf4	41.9	6.1	−6.87
NM_001145060	Glyatl3	19.6	3.0	−6.55
NM_029336	1700022P22Rik	34.3	6.0	−5.75
NM_029911	Kcnk10	11.5	2.0	−5.64
NM_181345	Npm2	89.2	16.1	−5.53
NM_010060	Dnahc11	17.1	3.2	−5.26
NM_011409	Slfn3	189.8	37.0	−5.13
NM_028798	Crct1	16.4	3.3	−4.97
NM_183278	2200001I15Rik	1297.8	263.5	−4.92
NM_153553	Npas4	14.4	2.9	−4.92
NM_021311	Piwil1	14.9	3.1	−4.76
NR_036572		177.0	38.0	−4.66
NM_023785	Ppbp	39.6	9.0	−4.37
NM_138648	Olr1	152.6	37.7	−4.05
NM_001034964	Sorbs1	24.1	6.0	−4.03
NM_009513	Nrsn1	175.3	43.5	−4.02
NM_008380	Inhba	1460.8	363.0	−4.02
NM_177781	Trpa1	234.0	59.6	−3.93
NM_007560	Bmpr1b	69.1	17.6	−3.91
NM_008006	Fgf2	44.6	11.5	−3.87
NM_177337	Arl11	12.9	3.4	−3.86
NM_011410	Slfn4	119.9	31.7	−3.79
NM_001164466	Dpys	63.5	17.2	−3.7
NM_175456	Abra	33.8	9.2	−3.7
NM_013468	Ankrd1	404.0	110.6	−3.66
NM_008036	Fosb	140.1	39.4	−3.55
NM_001111060	Cd59a	15.2	4.3	−3.54
NM_153127	Mmrn2	1059.0	301.8	−3.51
NM_015759	Fgd3	84.5	24.1	−3.51
NM_001109914	Apold1	38.2	11.0	−3.46
NM_008741	Nsg2	41.5	12.0	−3.45
NM_026262	4930524B15Rik	56.2	16.4	−3.43
NM_008216	Has2	506.8	148.4	−3.41
NM_009606	Acta1	226.8	70.4	−3.22
NM_009922	Cnn1	769.5	264.8	−2.91
NM_177371	Tnfsf15	52.4	18.2	−2.88
NM_010137	Epas1	996.3	351.5	−2.83
NM_011198	Ptgs2	2347.5	837.5	−2.8
NM_008008	Fgf7	392.8	141.3	−2.78
NM_177027	Zcchc7	88.9	33.1	−2.69
NM_009378	Thbd	1202.5	454.8	−2.64
NM_177293	Mtap7d3	118.1	45.2	−2.61
NM_001081020	Adamts6	123.4	47.4	−2.6
NM_001081328	4833446K15Rik	70.0	27.5	−2.55
NM_008871	Serpine1	10707.5	4247.5	−2.52
NM_172118	Myl9	2142.5	848.0	−2.52
NM_001085378	Trpc4ap	187.3	483.8	2.58
NM_054085	Alpk3	630.8	1676.3	2.65
NM_030127	Htra3	10.7	28.9	2.69
NM_016968	Olig1	241.8	663.3	2.74
NM_009154	Sema5a	597.8	1667.5	2.79
NM_145981	Phyhip	59.7	167.5	2.81
NM_016967	Olig2	251.5	718.0	2.85
NM_019626	Cbln1	132.8	384.0	2.89
NM_028775	Cyp2s1	175.5	509.8	2.9
NM_028772	Dmgdh	24.1	70.0	2.9
NM_009760	Bnip3	1907.5	5652.5	2.96
NM_175290	Nlrp4f	480.0	1447.5	3.01
NM_139148	Clca4	17.4	52.4	3.01
NM_023566	Muc2	14.8	45.6	3.09
NM_177823	Ubash3a	40.2	127.9	3.17
NM_008681	Ndrg1	617.8	2073.3	3.35
NM_207229	Plac9	107.8	364.0	3.38
NM_177905	Piwil4	594.0	2049.0	3.45
NM_029525	Prex2	60.9	225.8	3.71
NM_026922	Atp2c2	20.8	82.0	3.95
NM_001031621	Abca17	3.6	14.9	4.13
NM_054049	Osr2	12.5	54.1	4.31
NM_025325	Haao	3.7	16.8	4.5
NM_011374	St8sia1	207.3	1069.0	5.15
NM_007745	Cort	2.2	11.2	5.15
NM_001098789	Ndufa4l2	55.8	288.8	5.18
NM_007575	Ciita	6.9	36.0	5.25
NM_001085541	RP23-231M4.1	5.0	27.3	5.44
NR_028307		1.4	9.2	6.63
NR_002891		2.0	13.4	6.68
NM_021308	Piwil2	895.3	7682.5	8.57
NM_001025388	Eno1	524.8	6220.0	11.9
NM_008169	Grin1	1.8	24.6	13.6
NM_172808	Antxrl	0.0	4.6	Inf

## Discussion

In this study, we determined that PIWI protein family is dispensable for mouse somatic development and for reprogramming of fibroblasts into pluripotent stem cells. This is in contrast to the essential role of PIWI proteins for embryogenesis and somatic development in some non-mammalian animals. For example, in planarians and ascidians, PIWI homologs are necessary for the function of pluripotent stem cells that are capable of giving rise to all three germ layers during whole body regeneration [27]. In *Drosophila*, *piwi* functions in epigenetic and post-transcriptional gene regulation and in influencing somatic cell functions [44], [45]. Furthermore, it mediates canalization, a molecular mechanism that buffers the impact of genotypic and environmental variations on phenotype, to enhance developmental robustness [46]–[48]. Embryos depleted of maternal PIWI proteins display various severe mitotic defects including abnormal nuclear morphology, cell cycle arrest, and asynchronous nuclear divisions [35]. In mammals, the somatic role of PIWI proteins has not been explored sufficiently. HIWI was described in human bone marrow CD34+ hematopoietic stem cells (HSCs) and progenitors. However, we recently reported that mice lacking all PIWI protein family members were able to maintain long-term hematopoiesis with no observable effect on the homeostatic HSC compartment, suggesting PIWI is dispensable for normal HSC function [49]. The iPSCs lacking all PIWI proteins exhibited pluripotent gene expression and teratoma formation, all of which were indistinguishable from their littermate control cells. Moreover, unlike the critical role of *piwi* in the maternal-to-zygotic transition in *Drosophila*, ovaries lacking all PIWI proteins are functional and the female mice are fertile. The resulting ovaries were capable of supporting embryonic development and generated viable pups that survive to adulthood, suggesting that there might be compensatory mechanisms such as endo-siRNA to compensate for the function of the piRNA-PIWI pathway in murine oogenesis. Taken together, our results show that mammalian PIWI functions are restricted to the male germ line. Our study cannot rule out the possibility that the lack of PIWI protein family members could affect the mice as they age. In addition, their ability to cope with tissue damage caused by genetic and environmental stress could be compromised. Likewise, we cannot rule out minor effects of PIWI proteins on the differentiation capacity of reprogrammed iPSCs. These potential roles await further analysis.

## Materials and Methods

### Ethics statement

Animal experiments in this study were carried out in accordance with the Animal Use Protocols as approved by the Institutional Animal Care and Use Committee, Yale University (IACUC Protocol number: 2009–11087).

### Generation of *Miwi* ^-/-^ *Mili* ^-/-^ *Miwi2* ^-/-^ *Oct-GFP* MEFs

To avoid functional redundancy between PIWI family members, we generated cells completely deficient in *Miwi, Mili*, and *Miwi2* to make a TKO mutant [29], [31], [32]. To generate TKO MEFs with the *Oct-GFP* reporter, *Miwi^-/-^Mili^-/-^* female mice were crossed to *Oct4-GFP* reporter mice [36] (a kind gift from Dr. Valentina Greco, Yale University). The *Miwi^+/-^Mili^+/-^* Oct4-GFP^+/-^ were then crossed with *Miwi2^+/-^* mice (a kind gift from Dr. Haig Kazazian, The Johns Hopkins University School of Medicine) to generate *Miwi^+/-^Mili^+/-^Miwi2^+/-^ Oct4-GFP ^+/-^*mice. Male and female triple-heterozygotes with *Oct4-GFP ^+/-^* were intercrossed to generate female TKO Oct4-GFP homozygous mice. To generate Piwi TKO embryos, MEFs were derived from day 12.5–17.5 postcoitum embryos by breeding *Miwi^+/−^ Mili^+/−^ Miwi2^+/-^ Oct4-GFP* homozygous male mice to *Oct4-GFP* homozygous females, either triple or double knockouts of *Piwi*. Triple-heterozygotes were derived from littermate embryos and served as controls. Gonads, head, and internal organs were removed before processing the embryos for MEF isolation. The embryos used in this study are of mixed C57BL/6-s129 background. All the MEFs were maintained in D-MEM (high glucose) supplemented with 10% fetal bovine serum (Life Technologies), 0.1 mM MEM Non-Essential Amino Acids (Life Technologies), 6 mM L-glutamine (Life Technologies), 1 mM MEM Sodium Pyruvate (Life Technologies), and 1% Pen-Strep (Life Technologies) and were used between 2–3 passages for reprogramming experiments.

### Retroviral production, infection and iPSC generation

The following plasmids were obtained from Addgene: pMXs-Oct3/4-IP (15918), pMXs-Sox2-IP (15919), pMXs-Klf4-IP (15920) and pMXs-c-Myc-IP (16921). (Addgene). 293FT cells (Life Technologies) were maintained in D-MEM (high glucose) supplemented with 10% fetal bovine serum (Life Technologies), 0.1 mM MEM Non-Essential Amino Acids (Life Technologies), 6 mM L-glutamine (Life Technologies), 1 mM MEM Sodium Pyruvate(Life Technologies), and 1% Pen-Strep. For retrovirus production, the cells were seeded at 2×10^7^ cells per 175-cm flask a day before transfection. Immediately before transfection, the medium was replaced with 18 ml of D-MEM. A total of 28 µg DNA mixture containing 14 µg of plasmid DNA carrying the transgene (Oct4, Sox2, Klf4 or c-Myc) along with 1.4 µg of the envelope plasmid pVSV-G (a kind gift of Dr. In-Hyun Park, Yale University) and 12.6 µg of the packaging plasmid Gag-Pol (a kind gift of Dr. In-Hyun Park) was first diluted in 175 µl of 5% glucose (solution 1). 28 µl of 18 mM PEI (Polyethylenimine, Sigma) stock solution was then diluted in 175 µl of 5% glucose and placed on a vortex for 1 min (solution 2). After 10 min, both solutions were mixed, and the resulting solution was again placed on the vortex stirrer for 1 min. After 10 more minutes, the transfection mixture was completed up to 1 ml with D-MEM and added to the cells. After 6 h of incubation, the cells were supplemented with 1.8 ml of fetal calf serum. One day after transfection, medium was replaced with fresh complete medium. Retrovirus was harvested 48 h and 72 h post transfection by ultracentrifugation for 1.5 hours at 4 degrees at 47,810×g and store at −80°C.

We generated iPS cells as described by the Yamanaka group [50] with the following modifications: One day before transduction, MEFs were seeded at 4×10^4^ cells per well of a Matrigel coated 12-well plate. On the following day (considered day 0) the concentrated retrovirus were thawed and mixed with fresh MEF media up to 1 ml volume (per well) and 8 ng/ml polyprene, and then exposed to the MEFs at 37°C and 5% CO2. On day 1 the mixed viral supernatant was removed, the cells were washed twice with PBS and then cultured in fresh MEF medium. On day 2, the MEF medium was replaced with mESC medium containing D-MEM with 15% fetal bovine serum (Gemini Bio-Products), 0.1 mM MEM Non-Essential Amino Acids (Life Technologies), 6 mM L-glutamine (Life Technologies) 0.1 mM 2-mercaptoethanol (Sigma), 1% Pen-Strep (Life Technologies) and 10^3^ Uml^−1^ of LIF (Millipore-ESGRO). Thereafter, the medium was changed either every day or every other day, as required. In some experiments, treatment with 1 mM VPA begins on day 3 and lasts for a week [51]. mESC-like colonies started to appear about one week post-transduction. Colonies that were Oct4-GFP+ with mESC-like morphology were manually picked and transferred to 12-well plates pre-plated with mitomycin C-inactivated MEF feeders on day 16. Colonies that continued to expand and maintained their mESC-like morphology were further passaged; those that failed to expand and/or spontaneously differentiated were discarded.

In the iPSC growth competition assays, the S1B6A cell line was a generous gift from Dr. Natalia Ivanova.

### Flow cytometric analysis

SSEA-1 (eBioscience) and Sox2 (BD Pharmingen) staining was performed according to the manufacturer's instructions. Flow cytometric analysis was performed with an LSRII flow cytometer (BD Biosciences) and FlowJo software (TreeStar, Ashland, OR). Isotype controls were used in each experiment.

### Real time RT-PCR

Total RNA was isolated using the High Pure RNA Isolation Kit (Roche Diagnostics, Indianapolis, IN). For all RNA samples, genomic DNA was digested with RNase-free DNase I. cDNA was prepared using Superscript II Reverse Transcriptase (Life Technologies) with random primers (Life Technologies). Gene expression levels were determined using Applied Biosystems TaqMan Gene Expression Assays: *Miwi*: Mm00451649_m1; *Mili*: Mm01313483_m1; *Miwi2*: Mm01144775_m1; *HIWI*: Hs01041737_m1; *HILI*: Hs00216263_m1; *HIWI2*: Hs00381509_m1, and detected by iCycler iQ (Bio-Rad, Hercules, CA). Eukaryotic 18S rRNA: Hs99999901_s1 was used as an internal control for normalization. The qRT-PCR reactions were performed by absolute quantification method using individual Piwi plasmids as external calibration curve. The Taqman assay demonstrated specificity with individual Piwi with no detectable level of the other Piwi homologs.

### Teratoma formation assays

iPSCs were harvested and dissociated into single cells by trypsin. Following resuspension, 1×10^6^ cells were injected subcutaneously into five to eight-week-old SCID/Beige mice. Three to six weeks post-injection, teratomas were harvested and fixed overnight in 4% Paraformaldehyde, and sent to the Research Histology Facility at Yale School of Medicine for 5-µm paraffin sections and Hematoxylin and Eosin staining.

### Whole-transcriptome RNAseq analysis

Total RNA from individual iPS clones were isolated using the High Pure RNA Isolation Kit (Roche Diagnostics, Indianapolis, IN). For all samples, genomic DNA was digested with RNase-free DNase I. The RNA integrity (RNA Integrity Score≥8) and quantity was determined on the Agilent 2100 Bioanalyzer (Agilent; Palo Alto, CA, USA). Library preparation and sequencing were performed by the Yale Stem Cell Genomics Core Facility using the Illumina TruSeq RNA Sample Preparation kit. Samples were sequenced on an Illumina HiSeq 2000 using 50-cycle single-end sequencing. Four biological replicates were generated for the TKO and control, respectively.

Reads were mapped to Mus musculus (mm9) reference transcriptome from GenBank using BWA [52] alignment tool. Low-expressed genes that have less than 5 reads mapped in more than half of samples were excluded from further analysis. For sample-wise data normalization, we applied DESeq [53] using R statistical software. To achieve more reliable results, we applied 4 popular parametric or non-parametric methods for detecting differentially expressed genes (DEGs) using RNAseq data: edgeR [54], DESeq [53], baySeq [55], and RankProd [56]. Confident DEGs were identified that satisfy both of the following criteria: 1) in at least 2 methods with a False Discovery Rate (FDR) less than 0.05 (Benjamini-Hochberg method [57]) and 2) with an absolute fold change of 2.5 or greater.

Functional annotation was performed with the Database for Annotation, Visualization, and Integrated Discovery (David; http://david.abcc.ncifcrf.gov).

## Supporting Information

File S1 Table S1, Gene ontology analysis of down–regulated genes in triple knockout iPS cells. Table S2, Gene ontology analysis of up–regulated genes in triple knockout iPS cells.
(DOCX)
