# Supplementary material for: PIWI Proteins Are Dispensable for Mouse Somatic Development and Reprogramming of Fibroblasts into Pluripotent Stem Cells
Source: PLoS One. 2014 Sep 19;9(9):e97821. doi: 10.1371/journal.pone.0097821 (PMC4169525; doi:10.1371/journal.pone.0097821)
Supplement: File S1 — Table S1, Gene ontology analysis of down–regulated genes in triple knockout iPS cells. Table S2, Gene ontology analysis of up–regulated genes in triple knockout iPS cells. (DOCX) [file pone.0097821.s001.docx]

**Table S1:** Gene ontology analysis of down–regulated genes in triple knockout iPS cells.

| Biological process (BP) |  |  |  |
| --- | --- | --- | --- |
| Term | Count | P-Value | Fold Enrichment |
| GO:0010557~positive regulation of macromolecule biosynthetic process | 7 | 1.03E-03 | 5.7 |
| GO:0031328~positive regulation of cellular biosynthetic process | 7 | 1.27E-03 | 5.5 |
| GO:0009891~positive regulation of biosynthetic process | 7 | 1.34E-03 | 5.4 |
| GO:0010604~positive regulation of macromolecule metabolic process | 7 | 2.56E-03 | 4.8 |
| GO:0031325~positive regulation of cellular metabolic process | 7 | 2.79E-03 | 4.7 |
| GO:0009893~positive regulation of metabolic process | 7 | 3.73E-03 | 4.4 |
| GO:0045941~positive regulation of transcription | 6 | 3.89E-03 | 5.4 |
| GO:0010628~positive regulation of gene expression | 6 | 4.36E-03 | 5.3 |
| GO:0045935~positive regulation of nucleobase, nucleoside, nucleotide and nucleic acid metabolic process | 6 | 5.26E-03 | 5.1 |
| GO:0051173~positive regulation of nitrogen compound metabolic process | 6 | 5.99E-03 | 4.9 |
| GO:0042127~regulation of cell proliferation | 6 | 6.58E-03 | 4.8 |
| GO:0051239~regulation of multicellular organismal process | 7 | 7.03E-03 | 3.9 |
| GO:0048522~positive regulation of cellular process | 9 | 7.51E-03 | 2.9 |
| GO:0045944~positive regulation of transcription from RNA polymerase II promoter | 5 | 8.14E-03 | 6.0 |
| GO:0006357~regulation of transcription from RNA polymerase II promoter | 6 | 1.15E-02 | 4.2 |
| GO:0045893~positive regulation of transcription, DNA-dependent | 5 | 1.36E-02 | 5.2 |
| GO:0051254~positive regulation of RNA metabolic process | 5 | 1.39E-02 | 5.1 |
| GO:0048518~positive regulation of biological process | 9 | 1.53E-02 | 2.6 |
| GO:0006979~response to oxidative stress | 3 | 1.63E-02 | 14.9 |
| GO:0007275~multicellular organismal development | 12 | 1.80E-02 | 2.1 |
| GO:0007167~enzyme linked receptor protein signaling pathway | 4 | 2.30E-02 | 6.3 |
| GO:0030324~lung development | 3 | 2.57E-02 | 11.6 |
| GO:0030323~respiratory tube development | 3 | 2.66E-02 | 11.4 |
| GO:0044093~positive regulation of molecular function | 4 | 3.09E-02 | 5.6 |
| GO:0060541~respiratory system development | 3 | 3.16E-02 | 10.4 |
| GO:0032502~developmental process | 12 | 3.22E-02 | 1.9 |
| GO:0042221~response to chemical stimulus | 6 | 4.63E-02 | 2.9 |
| GO:0030036~actin cytoskeleton organization | 3 | 5.29E-02 | 7.8 |
| GO:0009888~tissue development | 5 | 5.34E-02 | 3.4 |
| GO:0009605~response to external stimulus | 5 | 5.39E-02 | 3.4 |
| GO:0031032~actomyosin structure organization | 2 | 5.48E-02 | 34.5 |
| GO:0030029~actin filament-based process | 3 | 5.93E-02 | 7.3 |
| GO:0008543~fibroblast growth factor receptor signaling pathway | 2 | 6.54E-02 | 28.7 |
| GO:0022603~regulation of anatomical structure morphogenesis | 3 | 6.60E-02 | 6.9 |
| GO:0050679~positive regulation of epithelial cell proliferation | 2 | 6.75E-02 | 27.8 |
| GO:0007169~transmembrane receptor protein tyrosine kinase signaling pathway | 3 | 6.91E-02 | 6.7 |
| GO:0040007~growth | 3 | 6.97E-02 | 6.7 |
| GO:0048732~gland development | 3 | 7.22E-02 | 6.6 |
| GO:0048513~organ development | 8 | 7.26E-02 | 2.1 |
| GO:0048731~system development | 9 | 7.54E-02 | 1.9 |
| GO:0051781~positive regulation of cell division | 2 | 7.80E-02 | 23.9 |
| GO:0050896~response to stimulus | 10 | 8.39E-02 | 1.8 |
| GO:0048477~oogenesis | 2 | 8.42E-02 | 22.1 |
| GO:0051302~regulation of cell division | 2 | 8.63E-02 | 21.5 |
| GO:0008285~negative regulation of cell proliferation | 3 | 9.01E-02 | 5.8 |
| GO:0045765~regulation of angiogenesis | 2 | 9.66E-02 | 19.2 |
|  |  |  |  |
| Cellular compartment (CC) |  |  |  |
| Term | Count | P-Value | Fold Enrichment |
| GO:0005576~extracellular region | 12 | 5.19E-04 | 3.2 |
| GO:0005615~extracellular space | 6 | 4.38E-03 | 5.3 |
| GO:0015629~actin cytoskeleton | 4 | 9.49E-03 | 8.8 |
| GO:0030017~sarcomere | 3 | 1.27E-02 | 17.0 |
| GO:0044449~contractile fiber part | 3 | 1.46E-02 | 15.8 |
| GO:0030016~myofibril | 3 | 1.62E-02 | 14.9 |
| GO:0043292~contractile fiber | 3 | 1.76E-02 | 14.3 |
| GO:0044421~extracellular region part | 6 | 2.35E-02 | 3.5 |
| GO:0001725~stress fiber | 2 | 4.82E-02 | 39.4 |
| GO:0032432~actin filament bundle | 2 | 5.03E-02 | 37.8 |
| GO:0042641~actomyosin | 2 | 5.64E-02 | 33.6 |
| GO:0005856~cytoskeleton | 6 | 8.89E-02 | 2.4 |
| GO:0005667~transcription factor complex | 3 | 8.94E-02 | 5.8 |
|  |  |  |  |
| Molecular Function (MF) |  |  |  |
| Term | Count | P-Value | Fold Enrichment |
| GO:0005102~receptor binding | 8 | 1.20E-03 | 4.7 |
| GO:0001664~G-protein-coupled receptor binding | 3 | 1.29E-02 | 16.9 |
| GO:0008083~growth factor activity | 3 | 4.72E-02 | 8.4 |
| GO:0005515~protein binding | 20 | 8.80E-02 | 1.4 |

**Table S2**: Gene ontology analysis of up–regulated genes in triple knockout iPS cells.

| Biological process (BP) |  |  |  |
| --- | --- | --- | --- |
| Term | Count | P-Value | Fold Enrichment |
| GO:0042391~regulation of membrane potential | 3 | 1.08E-02 | 17.9 |
| GO:0048699~generation of neurons | 4 | 2.71E-02 | 5.7 |
| GO:0022008~neurogenesis | 4 | 3.31E-02 | 5.3 |
| GO:0006873~cellular ion homeostasis | 3 | 4.67E-02 | 8.2 |
| GO:0055082~cellular chemical homeostasis | 3 | 4.90E-02 | 8.0 |
| GO:0032502~developmental process | 8 | 5.71E-02 | 2.1 |
| GO:0050801~ion homeostasis | 3 | 5.75E-02 | 7.3 |
| GO:0048663~neuron fate commitment | 2 | 6.10E-02 | 30.3 |
| GO:0048523~negative regulation of cellular process | 5 | 6.91E-02 | 3.0 |
| GO:0044238~primary metabolic process | 13 | 6.98E-02 | 1.5 |
| GO:0008152~metabolic process | 14 | 7.52E-02 | 1.4 |
| GO:0019725~cellular homeostasis | 3 | 7.58E-02 | 6.2 |
| GO:0009108~coenzyme biosynthetic process | 2 | 8.22E-02 | 22.2 |
| GO:0048878~chemical homeostasis | 3 | 8.44E-02 | 5.8 |
| GO:0048522~positive regulation of cellular process | 5 | 9.31E-02 | 2.7 |
| GO:0048519~negative regulation of biological process | 5 | 9.43E-02 | 2.7 |
| GO:0030182~neuron differentiation | 3 | 9.82E-02 | 5.3 |
|  |  |  |  |
| Molecular Function (MF) |  |  |  |
| Term | Count | P-Value | Fold Enrichment |
| GO:0030554~adenyl nucleotide binding | 7 | 1.41E-02 | 3.2 |
| GO:0001883~purine nucleoside binding | 7 | 1.47E-02 | 3.2 |
| GO:0001882~nucleoside binding | 7 | 1.52E-02 | 3.1 |
| GO:0017076~purine nucleotide binding | 7 | 3.45E-02 | 2.6 |
| GO:0005524~ATP binding | 6 | 4.09E-02 | 2.9 |
| GO:0032559~adenyl ribonucleotide binding | 6 | 4.28E-02 | 2.9 |
| GO:0016597~amino acid binding | 2 | 4.41E-02 | 42.4 |
| GO:0005488~binding | 20 | 6.51E-02 | 1.2 |
| GO:0000166~nucleotide binding | 7 | 6.59E-02 | 2.2 |
| GO:0046983~protein dimerization activity | 3 | 7.75E-02 | 6.2 |
| GO:0043176~amine binding | 2 | 8.00E-02 | 23.0 |
| GO:0032555~purine ribonucleotide binding | 6 | 8.91E-02 | 2.3 |
| GO:0032553~ribonucleotide binding | 6 | 8.91E-02 | 2.3 |
